# Supplementary material for: Aquila enables reference-assisted diploid personal genome assembly and comprehensive variant detection based on linked reads
Source: Nat Commun. 2021 Feb 17;12:1077. doi: 10.1038/s41467-021-21395-x (PMC7889865; doi:10.1038/s41467-021-21395-x)
Supplement: Supplementary file 1 — Supplementary Information [file 41467_2021_21395_MOESM1_ESM.pdf]

# Aquila: diploid personal genome assembly and comprehensive variant detection based on linked reads

Zhou et al.

## Supplementary Information

## Supplementary Tables and Figures

| Sequenced Library | Sample ID | Raw fold-coverage | $\mu_{FL}$ / W$\mu_{FL}$ (kb) | PCR duplication (%) | $C_F$ (X) |
|---|---|---|---|---|---|
| L1 | NA12878 | 103 | 79.0 / 304.3 | 19.97 | 123 |
| L2 | NA12878 | 192 | 24.0 / 41.1 | 10.92 | 334 |
| L3 | NA12878 | 106 | 99.2 / 214.5 | 11.09 | 958 |
| L7 | NA12878 | 78 | 37.4 / 82.3 | 4.84 | 243 |
| L4 | NA24385 | 100 | 120.8 / 267.4 | 18.51 | 208 |
| L5 | NA24385 | 100 | 64.2 / 151.7 | 12.39 | 803 |
| L6 | NA24385 | 100 | 92.1 / 216.9 | 10.88 | 1504 |

**Supplementary Table 1**. Key parameters of our 10x libraries from NA12878 and NA24385, L1 to L6, and a previously published library from 10x Genomics (L7).

| SNP | | True Positives | False Negatives | False Positives | Genotype Mismatch | Precision | Recall | F1 |
|---|---|---|---|---|---|---|---|---|
| **L1 (NA 12878)** | **Aquila** | 2,873,378 | 169,402 | 140,812 | 5,209 | 0.953 | 0.944 | 0.949 |
| | **FreeBayes** | 3,020,505 | 22,278 | 86,215 | 6,746 | 0.972 | 0.993 | 0.982 |
| | **Longranger** | 3,038,019 | 4,764 | 294,103 | 2,870 | 0.911 | 0.998 | 0.953 |
| **L2 (NA 12878)** | **Aquila** | 2,941,087 | 101,696 | 322,480 | 16,709 | 0.901 | 0.967 | 0.932 |
| | **FreeBayes** | 3,038,255 | 4,528 | 29,288 | 3,657 | 0.990 | 0.999 | 0.994 |
| | **Longranger** | 3,028,310 | 14,473 | 111,869 | 2,181 | 0.964 | 0.995 | 0.980 |
| **L7 (NA 12878)** | **Aquila** | 2,965,257 | 77,524 | 265,428 | 10,521 | 0.918 | 0.975 | 0.945 |
| | **FreeBayes** | 3,033,390 | 9,393 | 55,835 | 5,576 | 0.982 | 0.997 | 0.989 |
| **L4 (NA 24385)** | **Aquila** | 2,962,316 | 67,042 | 129,018 | 9,291 | 0.958 | 0.978 | 0.968 |
| | **FreeBayes** | 3,015,793 | 13,565 | 45,759 | 5,507 | 0.985 | 0.996 | 0.990 |
| | **Longranger** | 3,024,732 | 4,626 | 149,327 | 2,244 | 0.953 | 0.998 | 0.975 |
| **L6 (NA 24385)** | **Aquila** | 2,985,796 | 43,561 | 108,248 | 3,952 | 0.965 | 0.986 | 0.975 |
| | **FreeBayes** | 3,021,607 | 7,751 | 52,638 | 3,846 | 0.983 | 0.997 | 0.990 |
| | **Longranger** | N/A | | | | | | |

**Supplementary Table 2**. Accuracy of SNP calling, comparing assembly-based calling with two mapping-based approaches on the same libraries' linked read data. The benchmark is GiaB v3.3.2. Variant counts and performance scores were generated by RTGtools/hap.py, an Illumina haplotype comparison/benchmarking tool. Longranger calls were executed with "-vcmode=gatk". N/A, not applicable because Longranger could not complete runs in wgs mode.

| Small Indels | | True Positives | False Negatives | False Positives | Genotype Mismatch | Precision | Recall | F1 |
|---|---|---|---|---|---|---|---|---|
| NA12878 (hg38) | L1 | 475,455 | 55,927 | 67,656 | 27,554 | 0.875 | 0.895 | 0.885 |
| | L2 | 475,000 | 56,382 | 40,802 | 16,726 | 0.921 | 0.894 | 0.907 |
| | L3 | 499,301 | 32,081 | 40,292 | 9,493 | 0.925 | 0.940 | 0.932 |
| | L7 | 483,848 | 47,534 | 66,081 | 20,154 | 0.880 | 0.911 | 0.895 |
| NA24385 (hg38) | L4 | 469,972 | 33,081 | 59,299 | 11,365 | 0.888 | 0.934 | 0.911 |
| | L5 | 476,139 | 26,914 | 35,315 | 7,986 | 0.931 | 0.947 | 0.939 |
| | L6 | 474,306 | 28,747 | 36,944 | 8,353 | 0.928 | 0.943 | 0.935 |
| | L5+L6 | 473,895 | 29,158 | 15,724 | 5,335 | 0.968 | 0.942 | 0.955 |
| NA24385 (hg19) | L5 | 467,492 | 25,108 | 33,250 | 7,607 | 0.934 | 0.949 | 0.941 |
| | L5 | 465,488 | 27,112 | 34,674 | 8,037 | 0.931 | 0.945 | 0.938 |
| | L5+L6 | 467,624 | 24,976 | 14,512 | 4,777 | 0.970 | 0.949 | 0.959 |

**Supplementary Table 3**. Accuracy of Aquila small indel calls by benchmarking against the GiaB small indel callset v4.0.

| Library | Homo Aq | Homo Fb | Hetero Aq | Hetero Fb | Total Aq | Total Fb |
|---|---|---|---|---|---|---|
| L1 | 1,437,914 | 1,445,653 | 2,376,367 | 2,508,972 | 3,814,281 | 3,954,625 |
| L2 | 1,429,006 | 1,462,536 | 2,713,879 | 2,486,207 | 4,142,885 | 3,948,743 |
| L3 | 1,460,820 | 1,453,991 | 2,510,624 | 2,495,730 | 3,971,444 | 3,949,721 |
| L7 | 1,433,595 | 1,448,088 | 2,687,428 | 2,492,822 | 4,122,993 | 3,940,910 |
| L4 | 1,405,855 | 1,470,476 | 2,494,627 | 2,465,147 | 3,900,482 | 3,935,623 |
| L5 | 1,409,588 | 1,481,046 | 2,473,281 | 2,480,638 | 3,882,869 | 3,961,684 |
| L6 | 1,403,142 | 1,479,839 | 2,478,552 | 2,482,312 | 3,881,694 | 3,962,151 |

**Supplementary Table 4**. Comparison of the number of SNP calls, in diploid regions, using pairwise contig-to-reference alignment by Aquila (Aq) versus FreeBayes (Fb) calls on the LongRanger-aligned reads. Homo = homozygous, Hetero = heterozygous.

| Library | Deletions | | | Insertions | | |
|---|---|---|---|---|---|---|
| | Homo | Hetero | Total | Homo | Hetero | Total |
| **L1** | 116,003 | 387,479 | 503,482 | 115,136 | 374,926 | 490,062 |
| **L2** | 110,106 | 383,338 | 493,444 | 109,712 | 356,245 | 465,957 |
| **L3** | 131,602 | 385,741 | 517,343 | 130,004 | 359,966 | 489,970 |
| **L7** | 115,593 | 414,024 | 529,617 | 113,559 | 366,859 | 480,418 |
| **L4** | 125,019 | 394,529 | 519,548 | 124,243 | 379,019 | 503,262 |
| **L5** | 127,593 | 383,532 | 511,125 | 126,775 | 357,672 | 484,447 |
| **L6** | 125,692 | 382,402 | 508,094 | 125,042 | 355,661 | 480,703 |

**Supplementary Table 5**. Number of assembly-based small indel calls (< 50bp) in diploid regions for each library. Deletions: homozygous ones, heterozygous ones, and total number. Insertions: homozygous ones, heterozygous ones, and total number.

| Deletions | Homo | Hetero | Total | Insertions | Homo | Hetero | Total |
|---|---|---|---|---|---|---|---|
| **L1** | 2,427 | 13,953 | 16,380 | **L1** | 576 | 16,465 | 17,041 |
| **L2** | 3,072 | 13,222 | 16,294 | **L2** | 700 | 5,751 | 6,451 |
| **L3** | 3,398 | 14,408 | 17,806 | **L3** | 772 | 5,285 | 6,057 |
| **L7** | 2,849 | 15,922 | 18,771 | **L7** | 632 | 9,338 | 9,970 |
| **L4** | 2,952 | 14,962 | 17,914 | **L4** | 618 | 10,086 | 10,704 |
| **L5** | 3,254 | 14,131 | 17,385 | **L5** | 761 | 5,295 | 6,056 |
| **L6** | 3,294 | 14,594 | 17,888 | **L6** | 757 | 5,009 | 5,766 |

**Supplementary Table 6**. Number of SVs >= 50bp in diploid regions for each library. Deletions: homozygous ones, heterozygous ones, and total number. Insertions: homozygous ones, heterozygous ones, and total number.

| | SNPs (Initial phase) | SNPs (Inferred) | Indels (Inferred) | SVs (Inferred) | Total |
|---|---|---|---|---|---|
| **L1** | 1,963,497 | 578,558 | 698,828 | 29,795 | 3,270,678 |
| **L2** | 2,094,973 | 543,558 | 649,789 | 17,123 | 3,305,443 |
| **L3** | 1,746,930 | 802,233 | 674,018 | 18,895 | 3,242,076 |
| **L4** | 1,885,560 | 640,520 | 684,301 | 23,544 | 3,233,925 |
| **L5** | 1,973,507 | 538,224 | 660,832 | 18,373 | 3,190,936 |
| **L6** | 1,715,391 | 801,564 | 656,335 | 18,469 | 3,191,759 |

**Supplementary Table 7**. Number of phased variants for all libraries. Number of heterozygous SNPs phased by the Aquila haplotyping algorithm (SNPs, Initial phase), number of heterozygous SNPs phased by inference (SNPs, Inferred), number of heterozygous small Indels (< 50*bp*) phased by inference (Indels, Inferred), number of heterozygous SVs (>=50*bp*) phased by inference (SVs, Inferred), and the total number of heterozygous variants.

| Haplotype | Gene | ContigID | HG001 Truth | Called Genotype from Assembly | Components | Edit Distance Between Truth vs. Assembly |
|---|---|---|---|---|---|---|
| HP1 | HLA-A | 19355_PS91277:57577639_hp1_merge29991277:30086518_hp1-29791277:29891277_hp1-29891277:29991277_hp1 | A*01:01:01G | A*01:01:01G | exon2, exon3 | 0 |
| HP1 | HLA-B | 19364_PS91277:57577639_hp1_merge31591277:31691277_hp1-31487593:31591277_hp1-31386277:31487593_hp1-31291277:31386277_hp1-31191277:31291277_hp1 | B*08:01:01G | B*08:01:01G | exon2, exon3 | 0 |
| HP1 | HLA-C | 19364_PS91277:57577639_hp1_merge31591277:31691277_hp1-31487593:31591277_hp1-31386277:31487593_hp1-31291277:31386277_hp1-31191277:31291277_hp1 | C*07:01:01G | C*07:01:01G | exon2, exon3 | 0 |
| HP1 | HLA-DQA1 | 9978_PS32591277:32691277_hp1 | DQA1*05:01:01G | DQA1*05:01:01G | exon2 | 0 |
| HP1 | HLA-DQB1 | 19369_PS91277:57577639_hp1_merge32591277:32691277_hp1-32691277:32786277_hp1 | DQB1*02:01:01G | DQB1*02:01:01G | exon2 | 0 |
| HP2 | HLA-A | 9499_PS29891277:29991277_hp2 | A*11:01:01G | A*11:01:01G | exon2, exon3 | 0 |
| HP2 | HLA-B | 19826_PS91277:57577639_hp2_merge31291277:31386277_hp2-31386277:31487593_hp2 | B*56:01:01G | B*56:01:01G | exon2, exon3 | 0 |

| | | | | | | |
|---|---|---|---|---|---|---|
| HP2 | HLA-C | 19825_PS91277:57577639_hp2_merge31291277:31386277_hp2-31191277:31291277_hp2 | C*01:02:01G | C*01:02:01G | exon2, exon3 | 0 |
| HP2 | HLA-DQA1 | 19832_PS91277:57577639_hp2_merge32591277:32691277_hp2-32691277:32786277_hp2 | DQA1*01:01:01G | DQA1*01:01:01G | exon2 | 0 |
| HP2 | HLA-DQB1 | 19832_PS91277:57577639_hp2_merge32591277:32691277_hp2-32691277:32786277_hp2 | DQB1*05:01:01G | DQB1*05:01:01G | exon2 | 0 |
| HP2 | HLA-DRB1 | 8596_PS32486505:32591277_hp2 | DRB1*01:01:01G | DRB1*01:01:01G | exon2 | 0 |

**Supplementary Table 8.** HLA genotyping results and accuracy from library L3 (NA12878) by HLA*ASM [1].

| NA12878 | HLA-A | HLA-B | HLA-C | HLA-DQA1 | HLA-DQB1 | HLA-DRB1 |
|---|---|---|---|---|---|---|
| L1 | 1101/0101 | ../0801 | 0102/0701 | 0101/0501 | 0501/0201 | ../0101 |
| L2 | 1101/0101 | 5601/0801 | 0102/0701 | 0101/.. | ../.. | 0124<br>0101/0101 |
| L3 | 1101/0101 | 5601/0801 | 0102/0701 | 0101/0501 | 0501/0201 | ../0101 |

**Supplementary Table 9.** HLA genotyping summary from libraries L1, L2, and L3 (NA12878) by HLA*ASM. “..” refers to a missed call.

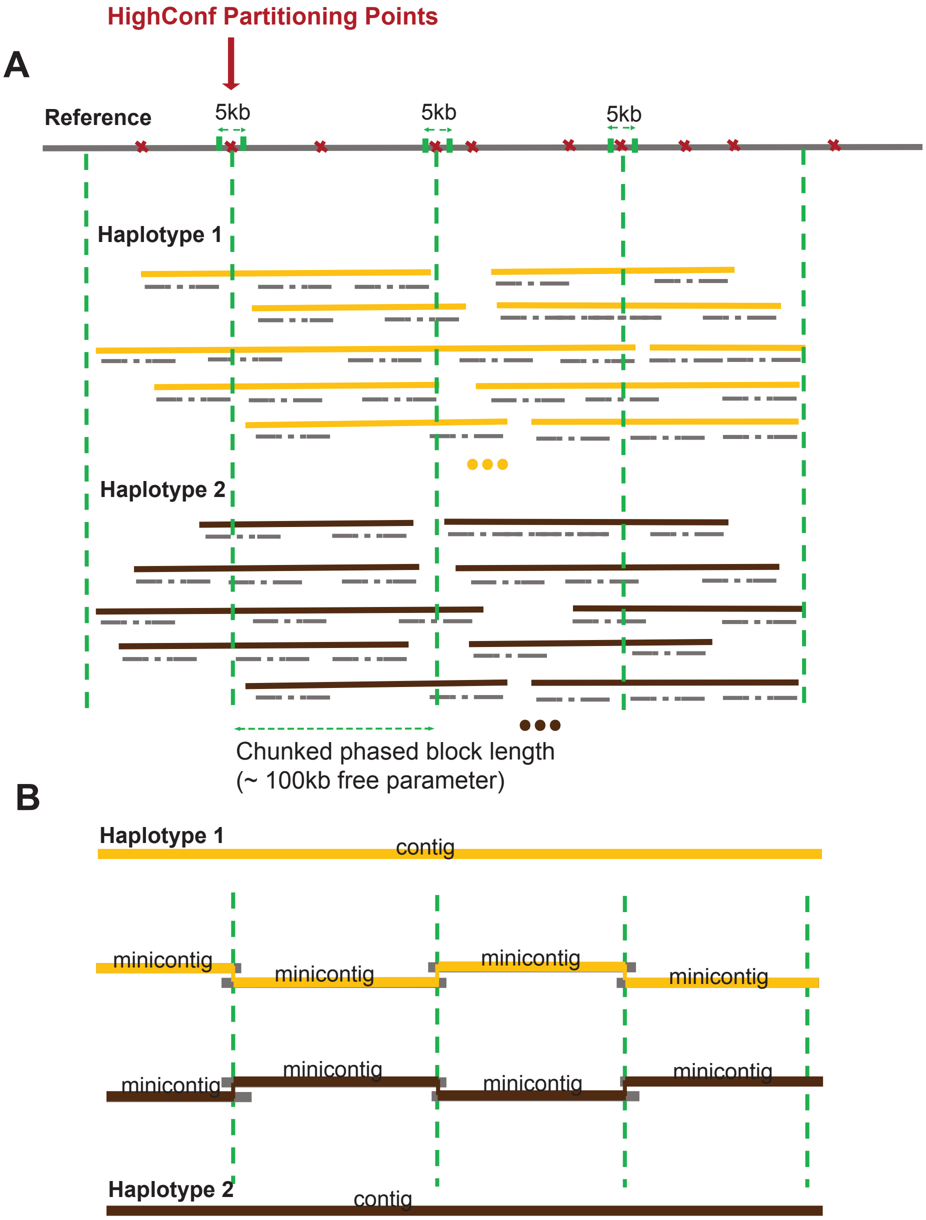


**Supplementary Figure 1**: High confidence partitioning points. A. Cutting one big phase block (in this example, ~ 300kb) from each haplotype into three small chunks to assemble "minicontigs" (in this example, ~100kb each) based on the high-confidence partitioning profile. There is a 5kb shift tolerance to allow Aquila find the high confidence partitioning point. B. Concatenation of minicontigs from each small chunk into final contig for both haplotypes within one big phase block.

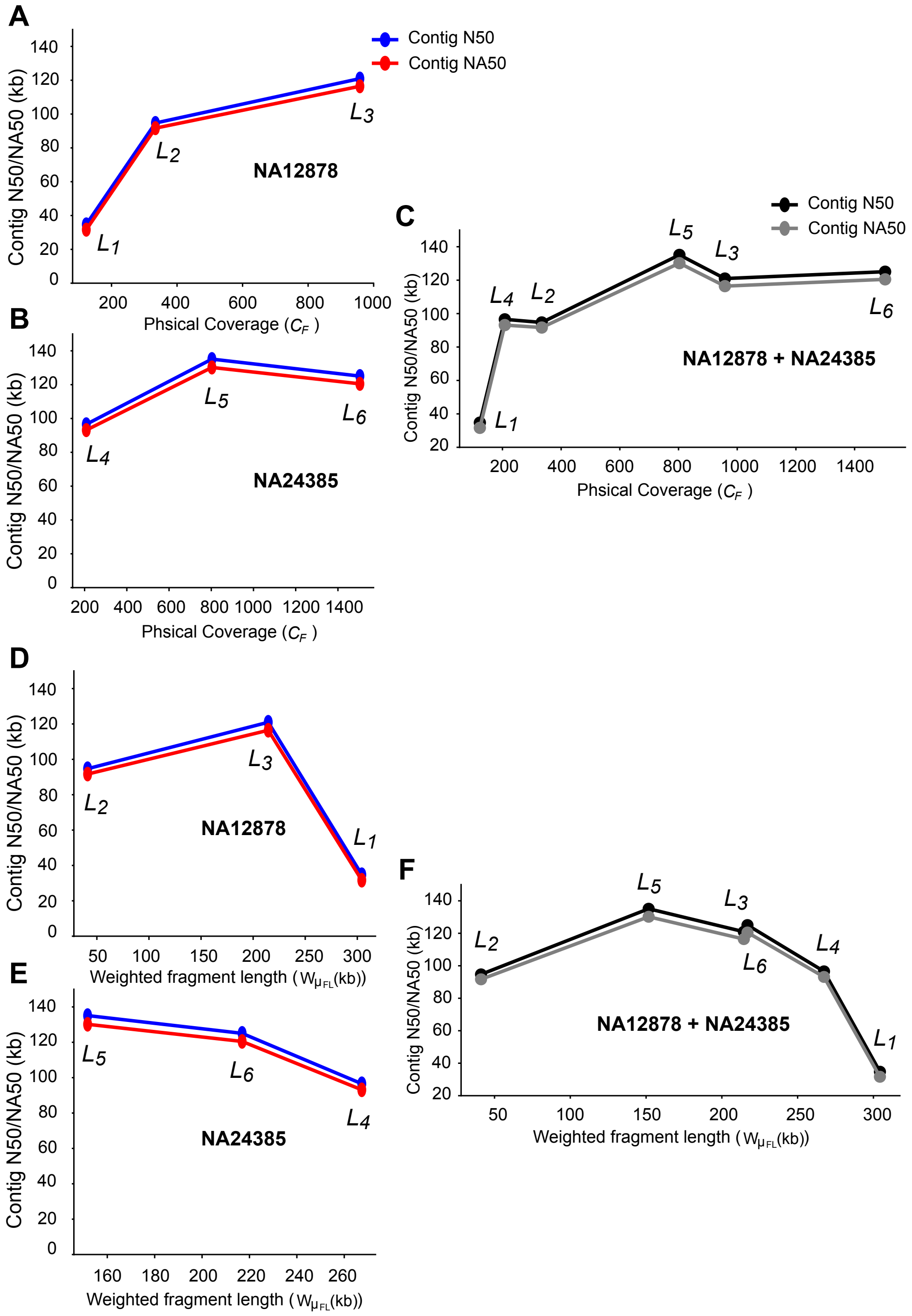


**Supplementary Figure 2**: A, B, C. Contig lengths as a function of physical coverage ($C_F$); D, E, F. Contig lengths as a function of weighted fragment length ($W_{\mu FL}$).

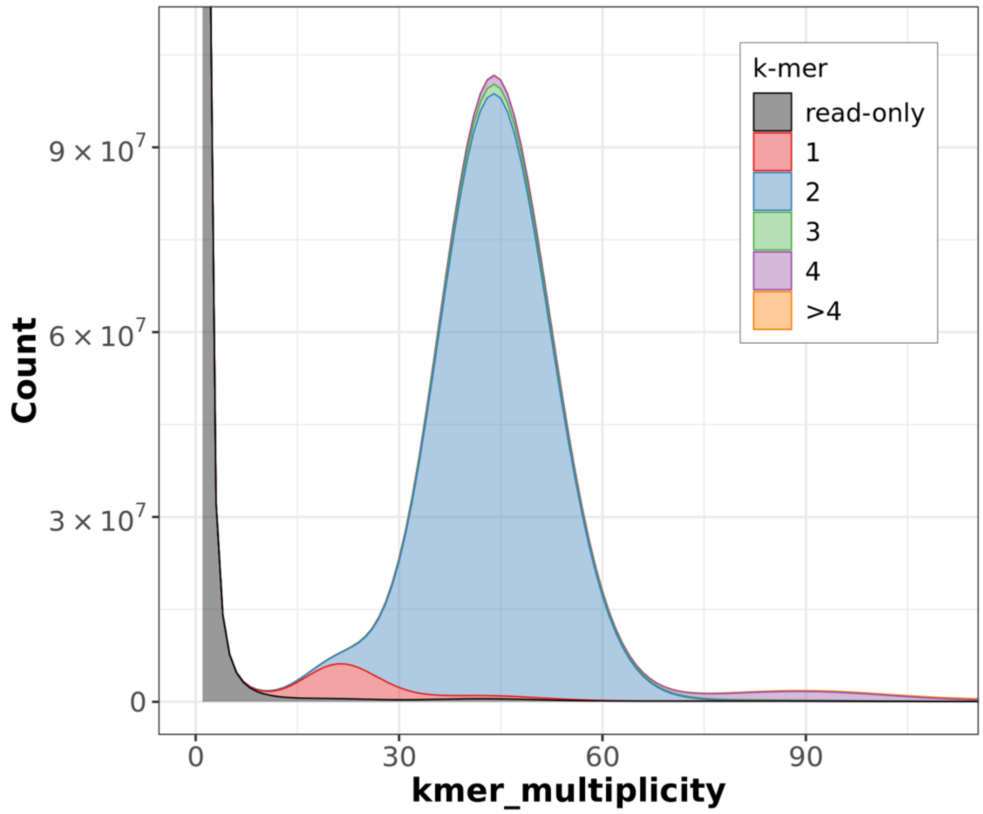


**Supplementary Figure 3**. Merqury copy number spectrum plots for haploid and diploid assemblies of L3 (NA12878). Copy number spectrum (spectra-cn) of the same k-mers plotted as stacked histograms colored by the copy numbers found in the L3 assemblies.

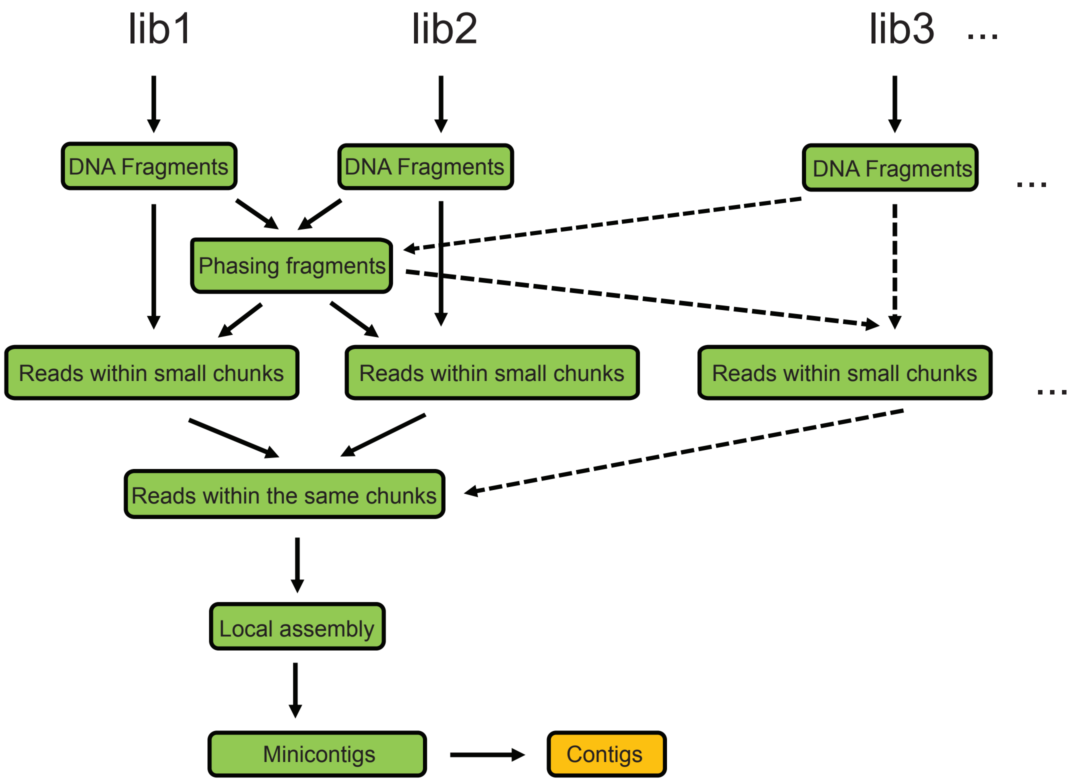


**Supplementary Figure 4**: Assemblies of multiple linked-read libraries. Long DNA molecules/fragments are first reconstructed from each library separately, but then the workflow is exactly the same as the single-library pipeline: The haplotyping module is applied to all the fragments together, short reads are extracted for each library based on the final phased information and then accumulated for the same chunks, followed by local assembly to generate minicontigs. Finally, minicontigs are iteratively concatenated into final contigs.

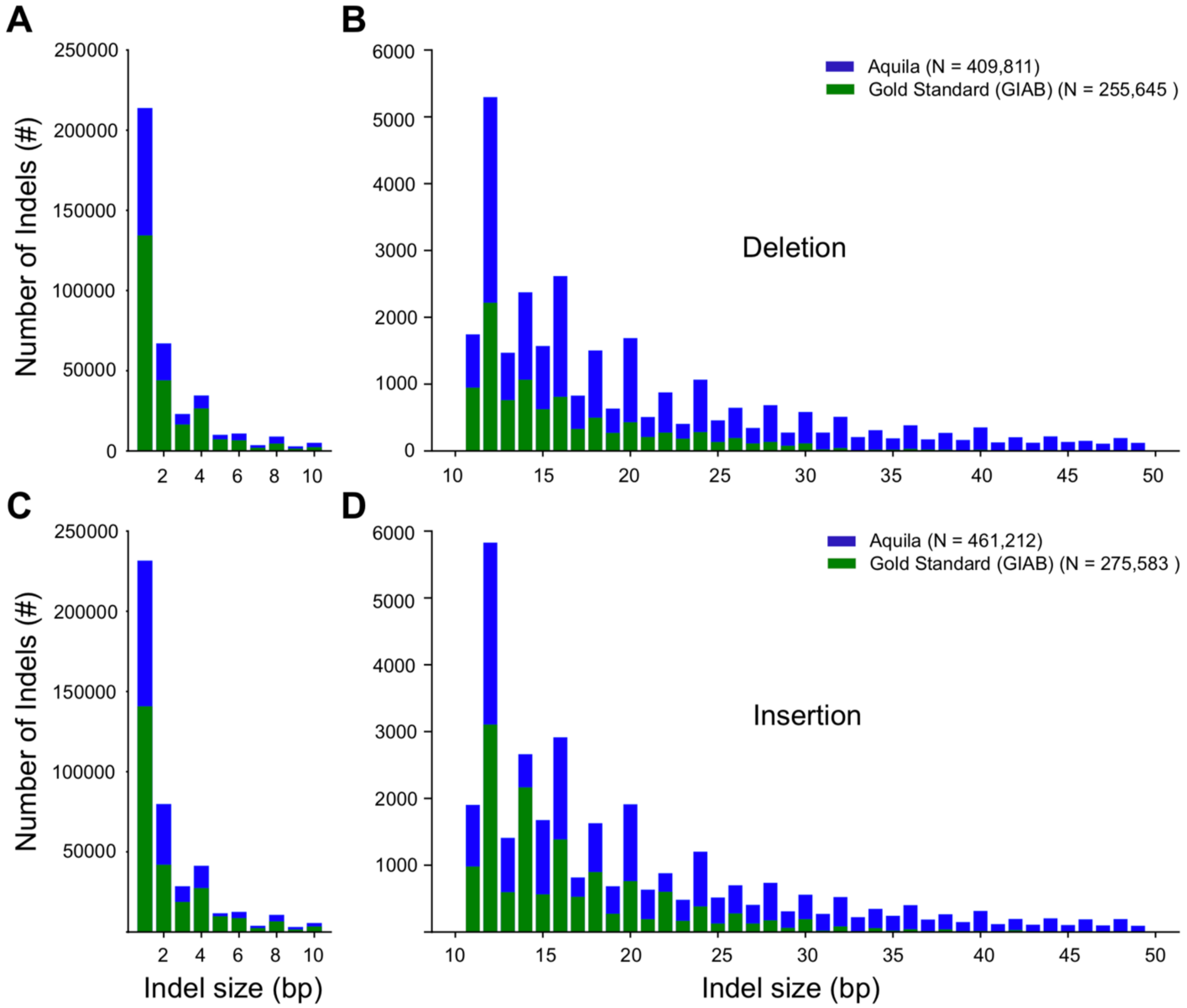


**Supplementary Figure 5**: Small indel size distribution of NA12878 for library 3 (blue plus green), with the distribution for GiaB benchmark in green only for comparison. A, deletions (<= 10bp); B, deletions (> 10bp and < 50bp); C, insertions (<= 10bp); D, insertions (> 10bp and < 50bp).

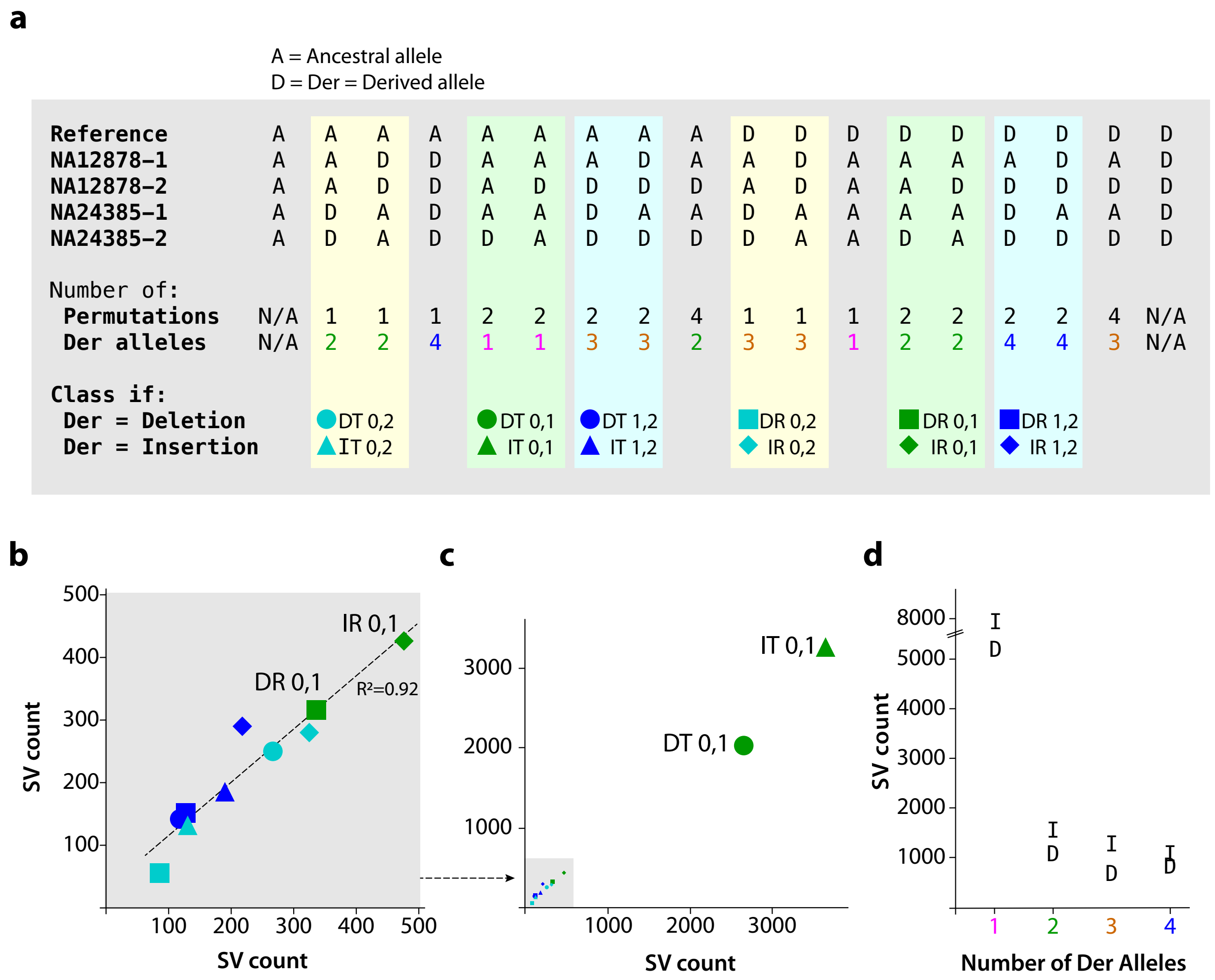


**Supplementary Figure 6**. Frequencies and genetic configurations of detected actual insertions and deletions as determined by alignment to ape genomes. (a) Each column depicts a possible configuration of ancestral and derived alleles in the five haplomes; all possibilities are shown but we cannot detect variation shown in the first and last columns as all sequences are identical in those cases. Each pattern is distinct but, due to the nature of sampling, represents between 1 and 4 possible arrangements. The number of derived alleles in each pattern is also indicated. Shorthand for symbols indicating the molecular type and who carries the derived alleles (MD) are: DT, deletion carried by a target; DR, deletion carried by the reference; IT, insertion carried by a target, IR, insertion carried by the reference. 0,1: single heterozygote; 0,2: single homozygote; 1,2: one target is a heterozygote, the other a homozygote. (b,c) MD numbers in the individuals

correlated to each other. Symbols represent classes as described in (a). For example, the green diamond (IR 0,1) indicates SVs defined by the fact that the derived allele is an insertion ("I") in the reference sequence ("R"), designated as "IR". There are 475 SVs in which NA12878 is homozygous for the reference allele and NA24385 is heterozygous for the derived allele, and 428 SVs for which NA12878 is heterozygous for the derived allele and NA24385 is homozygous for the reference allele. All other data points are analogous, always comparing NA12878 calls to their independent and equivalent counterparts.

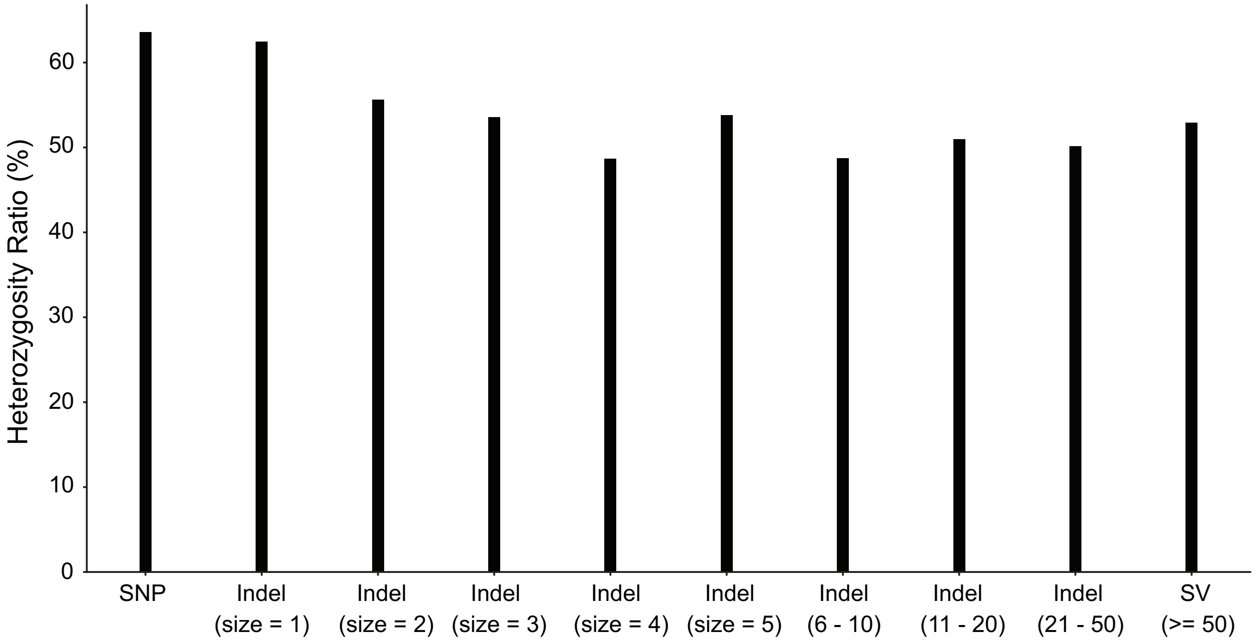


**Supplementary Figure 7**: Average heterozygosity of all types and sizes of discovered variants.

## Phase Inference

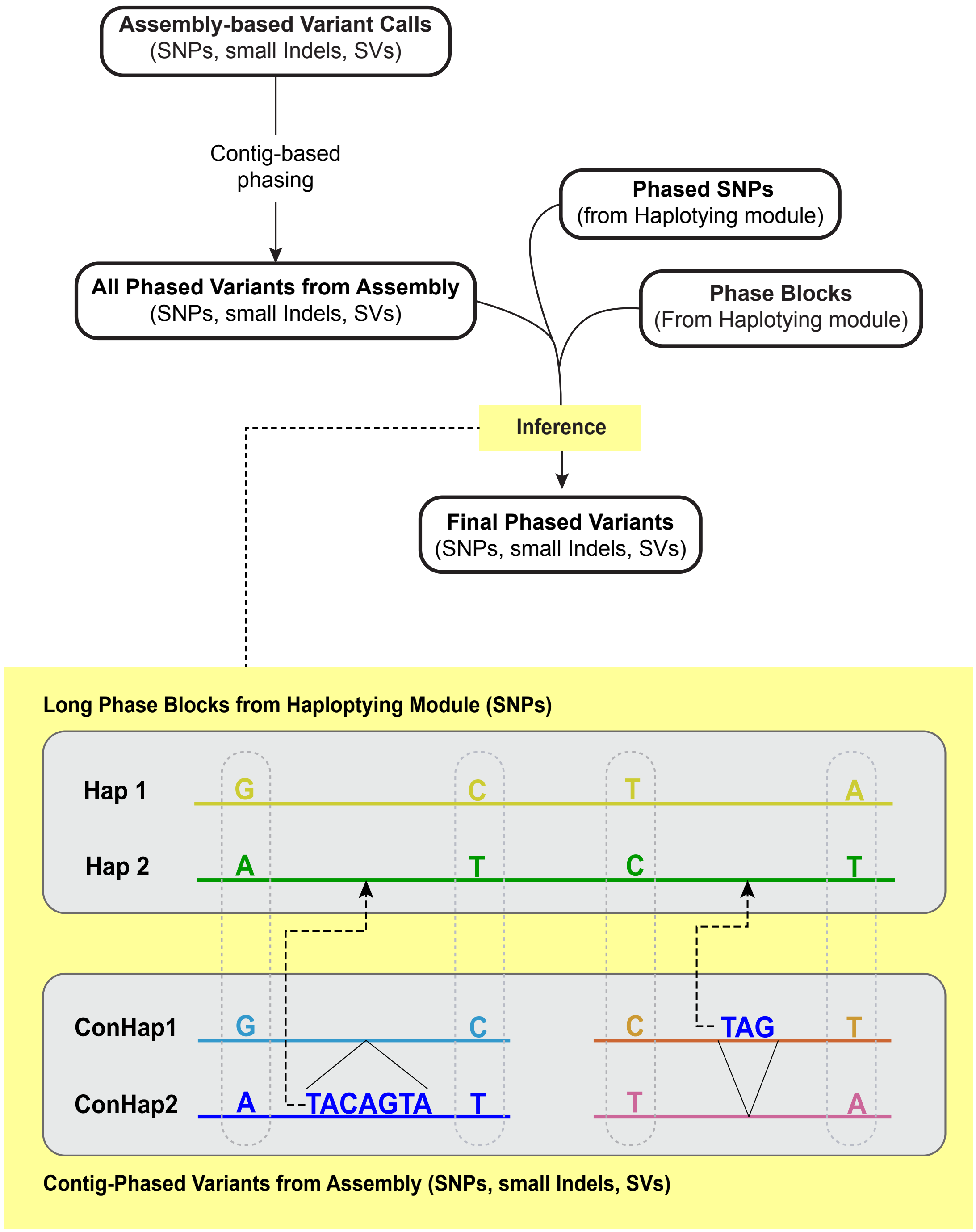


**Supplementary Figure 8**: Inference of phase for assembly-discovered variants on the basis of phase blocks generated in the Haplotyping module.

## SUPPLEMENTARY REFERENCES

1. Dilthey, A. T. *et al.* High-Accuracy HLA Type Inference from Whole-Genome Sequencing Data Using Population Reference Graphs. *PLoS Comput. Biol.* **12**, 1–16 (2016).
